# Celastrol Stimulates Hypoxia-Inducible Factor-1 Activity in Tumor Cells by Initiating the ROS/Akt/p70S6K Signaling Pathway and Enhancing Hypoxia-Inducible Factor-1α Protein Synthesis

**DOI:** 10.1371/journal.pone.0112470

**Published:** 2014-11-10

**Authors:** Xiaoxi Han, Shengkun Sun, Ming Zhao, Xiang Cheng, Guozhu Chen, Song Lin, Yifu Guan, Xiaodan Yu

**Affiliations:** 1 Department of Biochemistry and Molecular Biology, China Medical University, Shenyang, China; 2 Department of Urology, The General Hospital of PLA, Beijing, China; 3 Department of Cognitive Science, Institute of Basic Medical Sciences, Cognitive and Mental Health Research Center, Beijing, China; Maastricht University, Netherlands

## Abstract

Celastrol, a tripterine derived from the traditional Chinese medicine plant *Tripterygium wilfordii* Hook F. (“Thunder of God Vine”), has been reported to have multiple effects, such as anti-inflammation, suppression of tumor angiogenesis, inhibition of tumor growth, induction of apoptosis and protection of cells against human neurodegenerative diseases. However, the mechanisms that underlie these functions are not well defined. In this study, we reported for the first time that Celastrol could induce HIF-1α protein accumulation in multiple cancer cell lines in an oxygen-independent manner and that the enhanced HIF-1α protein entered the nucleus and promoted the transcription of the HIF-1 target genes VEGF and Glut-1. Celastrol did not influence HIF-1α transcription. Instead, Celastrol induced the accumulation of the HIF-1α protein by inducing ROS and activating Akt/p70S6K signaling to promote HIF-1α translation. In addition, we found that the activation of Akt by Celastrol was transient. With increased exposure time, inhibition of Hsp90 chaperone function by Celastrol led to the subsequent depletion of the Akt protein and thus to the suppression of Akt activity. Moreover, in HepG2 cells, the accumulation of HIF-1α increased the expression of BNIP3, which induced autophagy. However, HIF-1α and BNIP3 did not influence the cytotoxicity of Celastrol because the main mechanism by which Celastrol kills cancer cells is through stimulating ROS-mediated JNK activation and inducing apoptosis. Furthermore, our data showed that the dose required for Celastrol to induce HIF-1α protein accumulation and enhance HIF-1α transcriptional activation was below its cytotoxic threshold. A cytotoxic dose of Celastrol for cancer cells did not display cytotoxicity in LO2 normal human liver cells, which indicated that the novel functions of Celastrol in regulating HIF-1 signaling and inducing autophagy might be used in new applications, such as in anti-inflammation and protection of cells against human neurodegenerative diseases. Future studies regarding these applications are required.

## Introduction

Hypoxia-inducible factor 1 (HIF-1) is the key regulator of the hypoxia response. HIF-1 is a heterodimer composed of HIF-1α and HIF-1β [1]. Unlike the constitutively expressed HIF-1β, HIF-1α is induced by hypoxia, and this oxygen-sensitive induction occurs by decreasing protein degradation instead of enhancing mRNA expression. In normoxia, the HIF-1α protein is barely detectable because the Von Hippel Lindau gene (VHL) mediates its ubiquitination and rapid degradation through the proline hydroxylases (PHDs) and the proteasome pathway. The activities of PHDs are dependent on oxygen, and the binding of pVHL to HIF-1α requires the PHD-mediated modification of the oxygen-dependent degradation domain (ODD) of the protein. Therefore, HIF-1α cannot be hydroxylated and degraded during hypoxia [2]. In hypoxic circumstances, HIF-1α accumulates, translocates to the nucleus and binds to HIF-1β to form the active transcription factor HIF-1. The HIF-1 complex then binds to hypoxia response element (HRE) sequences in the promoters of HIF-1 target genes to initiate gene expression [1]. Many genes regulated by HIF-1α are involved in glycolysis, glucose metabolism, mitochondrial function, angiogenesis, cell survival, apoptosis and resistance to oxidative stress. In this regard, HIF-1 activation may play different roles in triggering cellular protection and metabolic alterations because of the consequences of oxygen deprivation or apoptosis in the presence of different environmental factors.

Celastrol, a triterpenoid from the Celastracae family that is extracted from the plant *Tripterygium Wilfordii* [3], has been reported to have multiple biological functions. In addition to treating autoimmune and neurodegenerative diseases by its anti-oxidative and anti-inflammatory effects [4], [5], Celastrol is frequently investigated for its potential anti-cancer activities *in vitro* and *in vivo*, including inhibiting tumor cell proliferation, inducing apoptosis in different types of cancer cells [6]–[9] and synergistically enhancing the cytotoxicity of radiotherapy and some chemotherapeutic agents [10]–[13]. Although previous studies have reported that Celastrol has the potential to inhibit HIF-1α mRNA transcription and suppress hypoxia-induced angiogenesis and tumor metastasis [14], [15], in this study, we found that a short amount of time exposure of Celastrol did not affect the HIF-1α mRNA levels. Instead, we found that Celastrol could induce HIF-1α protein accumulation in multiple cancer cell lines in an oxygen-independent manner and that the enhanced HIF-1α protein entered the nucleus and promoted the transcription of the HIF-1 target genes VEGF and Glut-1. Celastrol induced the accumulation of the HIF-1α protein by inducing ROS, which initiates the activation of Akt/p70S6K signaling to promote HIF-1α translation. These new data indicate that the full effect and function of Celastrol in regulating HIF-1 signaling may require further evaluation.

## Materials and Methods

### Cell culture

The human hepatocarcinoma cell line HepG2, the cervical carcinoma cell line HeLa, the breast cancer cell line MCF-7, the prostate cancer cell line PC-3 and the non-small cell lung cancer cell line H1299 were obtained from the American Tissue Culture Collection (Manassas, VA, USA). The normal liver LO2 cell line was kindly offered by Dr. Yan Wang (Beijing Institute of Basic Medical Sciences ). The cells were cultured with Dulbecco’s modified Eagle medium (Gibco, Grand Island, NY, USA) containing 10% fetal bovine serum (FBS, HyClone, Logan, UT, USA). For the hypoxia experiments, the cells were cultured in a hypoxia chamber with 3% O_2_ and 5% CO_2_ (with the balance being N_2_). Alternatively, to mimic hypoxia, 100 µM Cobalt chloride (CoCl_2_) was added to the culture medium [16], and the cells were cultured for the indicated hours.

### Reagents and antibodies

Celastrol was purchased from Calbiochem (San Diego, CA, USA) and dissolved in DMSO. Cobalt chloride, N-acetylcysteine (NAC) and cycloheximide (CHX) were purchased from Sigma (St Louis, MO, USA). LY294002 was purchased from Alexis (San Diego, CA, USA). The antibodies used were as follows: anti-HIF-1α and anti-HIF-1β (BD Transduction Laboratories, San Jose, CA, USA); anti-Raf, anti-Akt, anti-p-Akt (S473), anti-p-p70S6K (T389) and anti-PARP (Cell Signaling, Beverly, MA, USA); anti-BNIP3, anti-p53 and anti-p21 (Santa Cruz, Dallas, TX, USA); and anti-Bcl2, anti Bcl-xL and anti-β-actin (Calbiochem, San Diego, CA, USA). The dual-Luciferase reporter assay system was purchased from Promega (Madison, WI, USA).

### Cell viability assay

Cell viability was analyzed using the MTT assay. The cells were seeded in a 96-well plate at a density of 1×10^4^ and were treated with the indicated concentrations of Celastrol for 6 or 24 h. After each time point, 20 µl MTT (0.5 mg/ml) was added to each well, and the cells were cultured for another 4 h. Then, the medium was removed and 100 µl DMSO was added. The absorbance was read using a microplate reader (Tecan Infinite F50).

### Cell cycle assay

HepG2 cells were collected after treatment with Celastrol for 24 h and then washed with PBS twice. The cells were fixed with 70% ethanol/PBS at −20°C overnight. After washing with PBS, the cells were treated with RNase for 30 min at 37°C, and the cell cycle distribution was analyzed by fluorescence-activated cell sorting (FACS) of propidium iodide-stained cells.

### Isolation of nuclei

Nuclei were isolated using a Nuclei Isolation Kit (Applygen Technologies Beijing, China) according to the manufacturer’s suggestions. Briefly, cells were collected by trypsinization and resuspended with the cytosol extraction reagent provided in the kit. The cells were homogenized using a grinder until less than 5% of the cells were intact, and the homogenate was centrifuged at 1000×g for 10 min at 4°C. The supernatant and pellet were retained as the cytosolic fraction and nuclear fraction, respectively. The nuclei were washed twice with the nuclear extraction reagent provided in the kit, centrifuged at 4,000×g for 5 min and then lysed with Laemmli buffer.

### Western blotting

For western blotting, the cells were washed with PBS and suspended in Laemmli Buffer (Bio-Rad Laboratories, Hercules, CA, USA). The protein concentration was quantified using a BCA Protein Assay Kit (Pierce, Rockford, IL, USA), and the proteins were separated by SDS-polyacrylamide gel electrophoresis (SDS-PAGE), with 50–100 µg of protein loaded into each lane, and then transferred to a polyvinylidene difluoride membrane (Bio-Rad). The membrane was blocked in 5% skim milk, blotted with primary antibodies for 12–15 h at 4°C and then incubated with a horseradish peroxidase-conjugated secondary antibody for 1 h at room temperature. The proteins were detected using a Super Enhanced Chemiluminescence Detection Kit (Applygen Technologies, Beijing, China).

### Transfection and luciferase assays

HepG2 cells were co-transfected with pGL3-HRE-Luc and pRL-CMV plasmids using Mega Tran 1.0 (OriGene, MD, USA) according to the suggested protocol. Twenty-four hours after transfection, the cells were treated with Celastrol for 6 h, and the luciferase activity was then measured using the Dual-Luciferase Reporter Assay System according to the manufacturer’s instructions.

### Real-time PCR

Total RNA was isolated from HepG2 cells using TRIzol Reagent (Invitrogen, Carlsbad, CA, USA) following a standard protocol. RNA was reverse transcribed into cDNA using the RevertAid First Strand cDNA Synthesis Kit (Fermentas, Glen Burnie, MD, USA) according to the manufacturer’s instructions, and the resulting cDNA was used for qRT-PCR reactions with SYBR Green PCR Master Mix (Fermentas) and the Stratagene Mx3000P QPCR System. The primers used for amplification were as follows:

VEGF sense primer, 5′-ATGAACTTTCTGCTGTCTTG-3′; VEGF antisense primer 5′- TGAACTTCACCACTTCGT −3′;

Glut-1 sense primer, 5′- GCTGTCTGGCATCAACGCTGTCTT −3′; Glut-1 antisense primer, 5′- GCCTGCTCGCTCCACCACAA −3′;

RPL13A sense primer 5′- CGCTCTGGACCGTCTCAA −3′; RPL13A antisense primer 5′- AGATAGGCAAACTTTCTTGTAGGC −3′.

Standard curve reactions and melt curves were routinely run to validate the primer pairs and PCR reactions. The expression of the genes of interest was normalized and analyzed using RPL13A as an internal reference according to the Pfaffl method [17].

### Measurement of intracellular ROS generation

Intracellular ROS generation was measured by flow cytometry with a 2′,7′-dichlorodihydrofluorescein diacetate (DCFH-DA) probe (Applygen Technologies, Beijing, China). Untreated or treated cells were stained with 20 µM DCFH-DA for 30 min in the dark and subsequently assayed by flow cytometry.

### Immunofluorescence microscopy

Cells cultured on glass coverslips were treated with Celastrol for the indicated time, fixed with 4% paraformaldehyde in PBS for 10 min at room temperature and permeabilized with PBS plus 0.5% Triton X-100 for 10 min. The cells were incubated with PBS containing 1% bovine serum albumin for 30 min at room temperature and then washed three times with PBS. The cells were labeled with different primary antibodies for 1 h at room temperature or overnight at 4°C, followed by a 1-h incubation with FITC-conjugated secondary antibodies. DNA was counterstained with DAPI or Hoechst 33258, and the coverslips were examined by fluorescence microscopy at 1000×magnification under an immersion oil lens with a Zeiss 510 META microscope.

### Small interfering RNA

The siRNAs for HIF1α (target sequence of 5′-AGTTATGATTGTGAAGTTA-3′) and BNIP3 (target sequence of 5′-TTCATGACGCTCGTGTTCCT-3′) and a control siRNA (target sequence of 5′-UUCUCCGAACGUGUCACGU-3′) were synthesized by Shanghai GeneChem (Shanghai, China), and siRNA knockdown was performed according to the manufacturer’s protocol. Aliquots of 2×10^5^ cells were plated in 6-well plates, incubated for 24 h and transfected with 100 nM target siRNA or control siRNA using Entranster-R (Engreen Biosystem, Beijing, China). After 24 h, the cells were treated with Celastrol for another 24 h, and the cells were collected and analyzed by western blotting.

### Statistical analysis

The results are presented as the means ± SD of at least three separate experiments. The differences between groups were evaluated by Student’s t-test or one-way analysis of variance (ANOVA) followed by Dunnett’s test for multiple comparisons. Analyses were done with GraphPad Prism software (Graphpad; La Jolla, CA, USA). The significance level was defined as * P<0.05, ** P<0.01, and *** P<0.001.

## Results

### Celastrol increases the protein level of HIF-1α in an oxygen-independent manner

**Figure 1:**
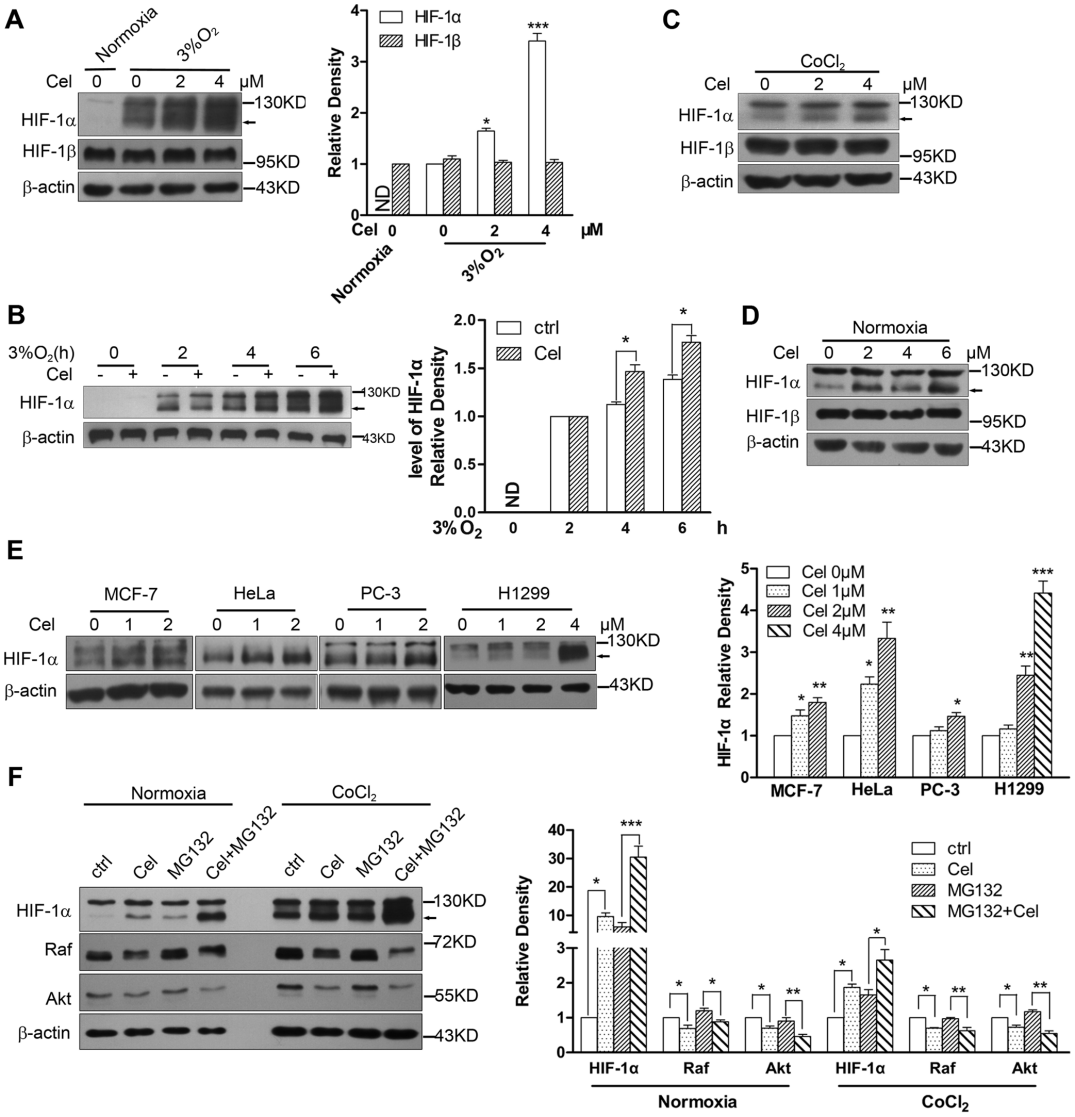
Celastrol increases the protein level of HIF-1α. 1a. Celastrol dose-dependently enhanced HIF-1α expression under hypoxia. HepG2 cells were cultured in either normoxia or 3% hypoxia and treated with the indicated doses of Celastrol for 6 h. Western blotting was used to detect the expression of HIF-1α and HIF-1β, and β-actin was used as a loading control; an arrow shows the position of HIF-1α. The histogram results are representative of the mean ± SD of three independent experiments. 1b. Celastrol time-dependently enhanced HIF-1α expression under hypoxia. HepG2 cells were cultured with 4 µM Celastrol for the indicated time in hypoxia. 1c. Celastrol enhanced HIF-1α expression in HepG2 cells under mimetic hypoxia induced by 100 µM CoCl_2_ and treated for 6 h. 1d. Celastrol dose-dependently enhanced HIF-1α expression under normoxia. HepG2 cells were treated with the indicated doses of Celastrol under normoxia for 6 h, and 100 µg of total protein was used for western blotting to detect HIF-1 proteins with a long exposure. 1e. Celastrol enhanced HIF-1α expression in multiple cell lines. MCF-7, HeLa, PC-3 and H1299 cells were treated with the indicated doses of Celastrol for 12 h under 3% hypoxia, and western blotting was used to detect the HIF-1 proteins. 1f. Celastrol decreased the levels of other Hsp90 client proteins but increased that of HIF-1α. HepG2 cells were treated with normal medium, 4 µM Celastrol for 6 h, 5 µM MG132 for 6 h or pretreated with MG132 for 1 h then treated with Celastrol for 6 h. Protein expression was determined by western blotting with the corresponding antibodies.

Unexpectedly, when we used Celastrol to treat HepG2 cells under 3% hypoxia for 6 h, we noticed that the protein level of HIF-1α increased in a time- and dose-dependent manner, but Celastrol did not affect the protein level of HIF-1β (Figs. 1a, 1b). Additionally, when using cobalt chloride (CoCl_2_) to mimic hypoxia, Celastrol also enhanced HIF-1α accumulation (Fig. 1c). More importantly, when the cells were treated with 2–6 µM Celastrol under normoxia, enhanced HIF-1α expression still occurred (Fig. 1d). The effect of Celastrol in enhancing HIF-1α expression was not cell-type specific because we also detected the same changes in human mammary carcinoma MCF-7 cells, cervical cancer HeLa cells, prostate cancer PC-3 cells and non-small cell lung cancer H1299 cells (Fig. 1e). These results supported the finding that Celastrol increased HIF-1α expression in an oxygen-independent manner. In addition, although the activation of the p53 gene has been reported to promote proteasomal degradation of the HIF-1α protein [18], the influence of p53 on this process was excluded because Celastrol could increase HIF-1α protein in MCF-7 cells, which have wild type p53, and in PC-3 and H1299 cells, which are p53 null. Interestingly, as an Hsp90 inhibitor, Celastrol could decrease the levels of Hsp90 client proteins, such as Raf and Akt from HepG2 cells; however, it increased the level of HIF-1α protein, though HIF-1α is also an Hsp90 client protein (Fig. 1f).

### Celastrol increases the HIF-1α protein level by enhancing its translation

**Figure 2:**
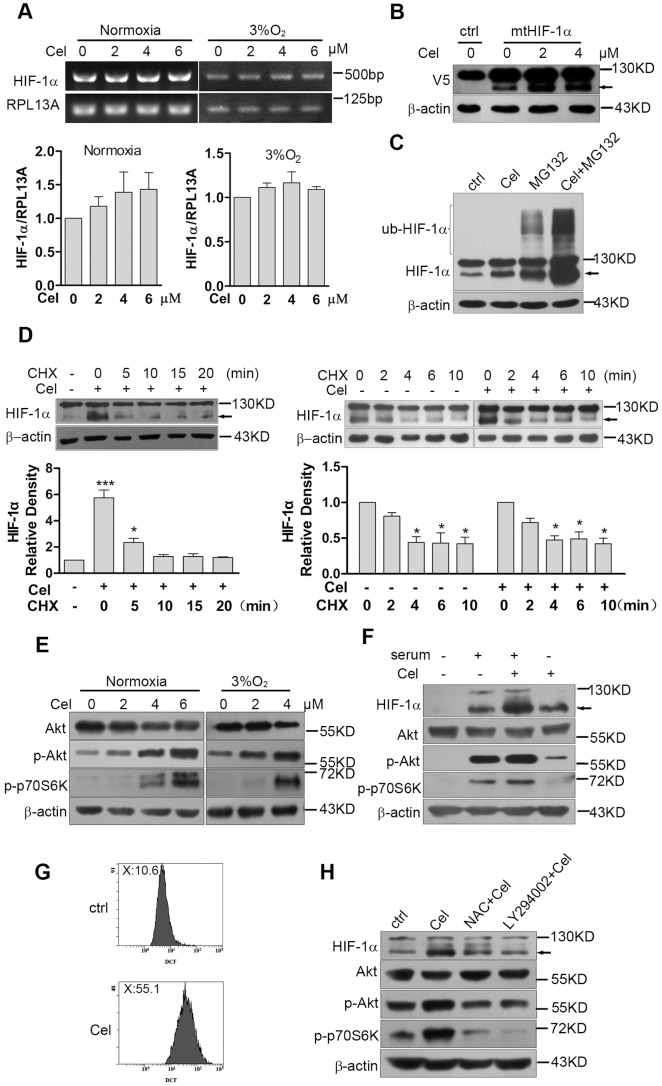
Celastrol increases the HIF-1α protein level by enhancing its translation. 2a. Celastrol did not affect HIF-1α transcription. After treatment with different concentrations of Celastrol for 6 h, total RNA was extracted, and the mRNA levels of HIF-1α and RPL13A were determined by RT-PCR. The relative expression of HIF-1α was normalized to that of RPL13A. The values are presented as the means ± SD of three independent experiments. 2b. Celastrol enhanced the expression of the HIF-1α P402A/P564A mutant. HeLa cells were seeded in 6-well plates and transiently transfected with either pcDNA-V5 empty vector (ctrl) or pcDNA3-P402A/P564A-HIF-1α-V5 (mtHIF-1). Twenty-four hours later, the cells were treated with 1–2 µM Celastrol for 6 h under normoxia. Mutant HIF-1α expression was analyzed by western blotting with the anti-V5 antibody. 2c. Celastrol did not affect HIF-1α ubiquitination. HepG2 cells were treated separately with 4 µM Celastrol, 10 µM MG132 or Celastrol plus MG132 for 4 h under normoxia, and the ub-HIF-1α protein level was determined by western blotting using the anti-HIF-1α antibody. 2d. The effect of the protein synthesis inhibitor CHX on Celastrol-induced HIF-1α accumulation. HepG2 cells were treated with or without 4 µM Celastrol for 6 h. Then, 10 µM CHX was added to the culture medium, and the cells were collected at the indicated times (left). HepG2 cells were cultured in medium containing 100 µM CoCl_2_ with or without 4 µM Celastrol for 4 h, then 10 µM CHX was added, and the cells were collected at the indicated times (right). The HIF-1α protein level was analyzed by western blot analysis. 2e. Celastrol induced AKT/p70S6K activation under normoxia and hypoxia. HepG2 cells were challenged with the indicated doses of Celastrol for 6 h under normoxia or hypoxia. The protein levels were analyzed by western blotting with the corresponding antibodies. 2f. Celastrol induced AKT/p70S6K activation under serum starvation. HepG2 cells were cultured in serum-free medium for 24 h. Then, 10% FBS, 4 µM Celastrol or both were added, and the cells were cultured for another 6 h. The protein expression was analyzed by western blotting with the corresponding antibodies. 2 g. Celastrol induced the accumulation of ROS. HepG2 cells were treated with 4 µM Celastrol for 12 h under normoxia. The levels of ROS were measured by DCFH-DA staining and subsequently assayed by flow cytometry. 2 h. The effect of Celastrol-induced HIF-1α accumulation depends on ROS-mediated AKT activation. HepG2 cells were pretreated with 5 mM NAC or 10 µM LY294002 for 1 h. Then, 4 µM Celastrol was added to the culture medium, and the cells were cultured for another 6 h. The protein expression was determined by western blotting with the corresponding antibodies.

Because it is known that the enhanced expression of HIF-1α under hypoxia is regulated at the protein level rather than at the mRNA level, we also excluded the possibility that Celastrol increased HIF-1α protein levels by promoting HIF-1α transcription, as Celastrol did not affect the mRNA expression of HIF-1α in HepG2 cells under normoxia or hypoxia (Fig. 2a). The most common mechanism for hypoxia-induced HIF-1α protein accumulation is mediated by prolyl hydroxylases (PHDs) that control HIF-1α by maintaining it at low levels under normal conditions; under hypoxia, PHDs activity drops and HIF-1α accumulates [2]. To investigate whether Celastrol increased the HIF-1α protein level by affecting PHDs activity, we transiently transfected the pcDNA3-V5 and pcDNA-P402/564A-HIF-1α-V5 vectors into HeLa cells separately. Because PHDs recognize the P402/564 sites of the HIF-1α protein, those sites were mutated to make the mutant HIF-1α protein resistant to PHD-VHL mediated degradation; therefore, the HIF-1α protein could be easily detected under normoxia. We then treated the cells with Celastrol, and the result showed that Celastrol still induced the accumulation of the mutated HIF-1α protein (Fig. 2b), which excluded the possibility that Celastrol enhanced HIF-1α expression by inhibiting PHDs. Previously, Celastrol has been identified as a proteasome inhibitor [19], [20]; therefore, we wondered whether Celastrol increased HIF-1α protein levels by enhancing its ubiquitination and blocking its degradation. We thus treated HepG2 cells separately with Celastrol, the proteasome inhibitor MG132 or Celastrol plus MG132 and detected the ubiquitination of HIF-1α by western blotting. The result showed that, unlike MG132, Celastrol alone did not increase the ubiquitination of HIF-1α, but Celastrol showed a remarkable, synergistic effect in enhancing MG132-induced ubiquitination of HIF-1α (Fig. 2c). These results excluded the possibility that Celastrol induced HIF-1α accumulation by promoting HIF-1α mRNA transcription or by inhibiting HIF-1α protein degradation. In addition, HIF-1α can be regulated at the translational level. Previous studies have reported that some types of stimulation, such as cytokines or serum, could upregulate the HIF-1α protein level via the activation of Akt/mTOR and inhibition of GSK3, which led to increased HIF-1α translation [21], [22]. To evaluate whether Celastrol enhanced HIF-1α protein levels via this mechanism, we first treated cells with or without Celastrol under normoxia and then added the protein synthesis inhibitor cycloheximide (CHX). The results showed that Celastrol-induced HIF-1α accumulation indeed required new protein synthesis because CHX could completely block this effect (Fig. 2d). Then, HepG2 cells were treated with 100 µM CoCl_2_ with or without 4 µM Celastrol for 4 h, and CHX was added to observe the degradation rate of HIF-1α. The result showed that Celastrol did not affect the degradation rate of the HIF-1α protein (Fig. 2d). Then, we treated HepG2 cells with Celastrol and confirmed that Celastrol dose-dependently induced the activation of Akt/p70S6K under normoxic and hypoxic conditions (Fig. 2e). In addition, we observed that Celastrol could induce the activation of Akt/p70S6K under serum starvation, which was also coincident with HIF-1α accumulation (Fig. 2f). Moreover, Celastrol induced ROS production (Fig. 2 g), which was coincident with the Celastrol-induced activation of Akt/p70S6K, and HIF-1α accumulation could be blocked by the PI3K inhibitor LY294002 and ROS scavenger NAC (Fig. 2 h). These results indicate that Celastrol may indirectly activate Akt by inducing ROS, and the activation of Akt further promotes HIF-1α translation.

### Celastrol promotes the hypoxia-induced, nuclear accumulation of the HIF-1α protein and increases the transcriptional activity of HIF-1α-target genes

**Figure 3:**
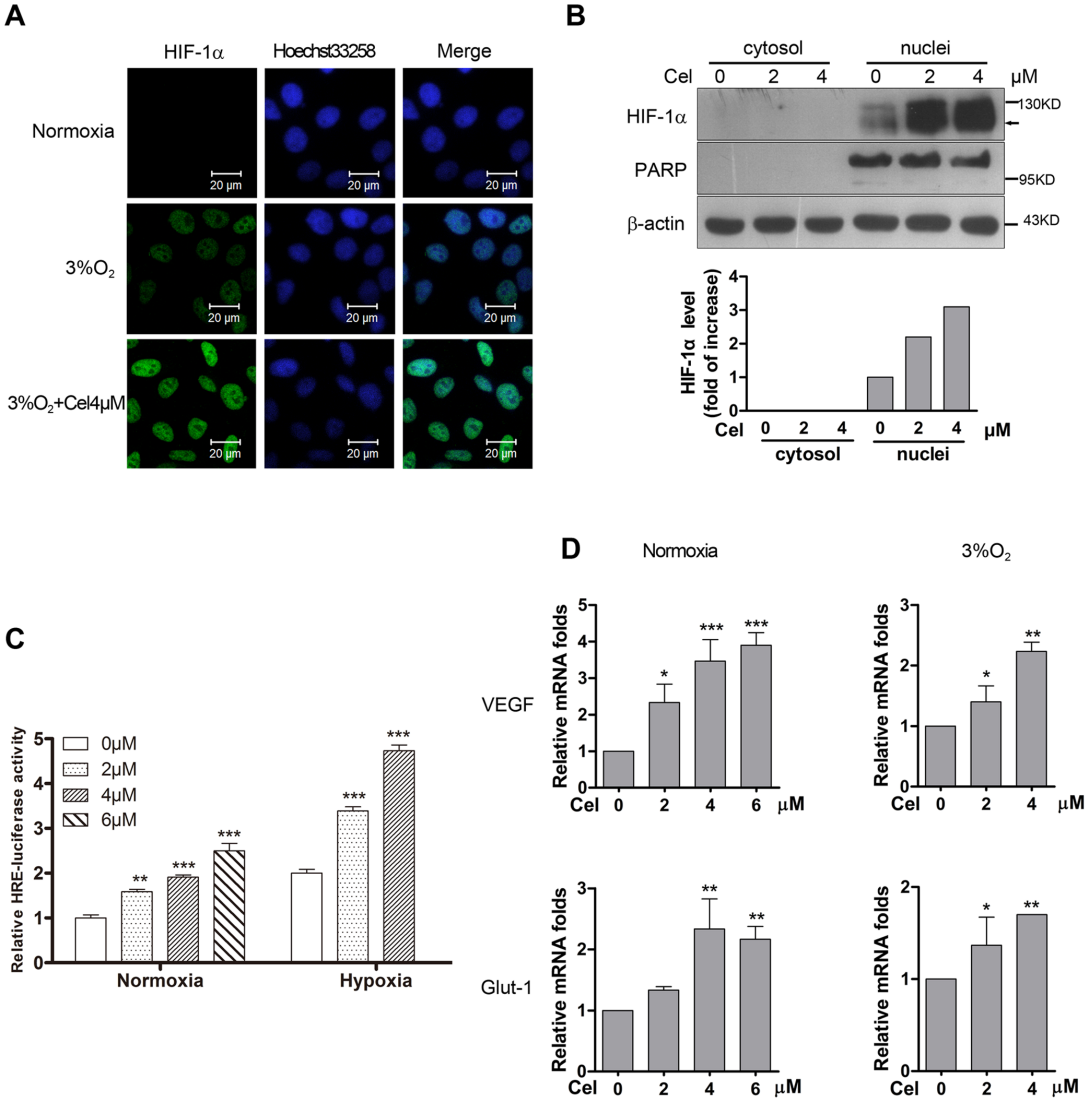
Celastrol promotes the hypoxia-induced accumulation of the HIF-1α protein in the nucleus, which increases the transcriptional activity of HIF-1α target genes. 3a. Celastrol enhances HIF-1α protein expression, which was localized in the nucleus. HepG2 cells were cultured in medium with or without 4 µM Celastrol for 6 h under normoxia or hypoxia. The subcellular localization of HIF-1α was determined by immunofluorescent staining using the anti-HIF-1α antibody,and the nucleus was immunolabeled with Hoechst 33258. 3b. HepG2 cells were treated with the indicated dose of Celastrol for 6 h, protein was extracted from the nucleus and cytosol, and the protein expression levels were revealed by western blot analysis. PARP served as the nuclear protein loading control. 3c. Celastrol promotes HIF-1α transcriptional activation activity. After transient transfection with the HRE-luciferase reporter plasmids for 24 h, HepG2 cells were challenged with the indicated doses of Celastrol in normoxia or hypoxia for another 6 h. Then, the HIF-1α transcriptional activation activity was analyzed by luciferase assay. The values are presented as the means ± SD of three independent experiments. 3d. Celastrol promotes the transcription of the HIF-1α target genes VEGF and Glut-1. The VEGF and Glut-1 mRNA levels were evaluated by real-time PCR. The values are presented as the means±SD of three independent experiments.

The effect of Celastrol in enhancing HIF-1α expression led us to further determine whether the enhanced expression of HIF-1α represented HIF-1 signal activation. First, we detected the cellular localization of the HIF-1α protein using immunofluorescent staining. As in Fig. 3a, immunofluorescent staining illustrated that the exposure of HepG2 cells to 3% hypoxia induced the HIF-1α protein to accumulate in the nucleus, and Celastrol-treated cells showed an obvious increase in HIF-1α protein levels in the nucleus. This result was further confirmed by detecting HIF-1α by western blotting in the cytosolic and nuclear fractions. As Fig. 3b shows, Celastrol-induced the nuclear localization of nearly all of the HIF-1α present in the cell, indicating that Celastrol did induce the activation of HIF-1α. This conclusion was reconfirmed using an HRE-luciferase assay to detect the transcriptional activation activity of HIF-1α and using real-time PCR to evaluate the transcriptional activity of its target genes, such as VEGF and Glut-1. The results showed that Celastrol oxygen-independently but dose-dependently enhanced the transcriptional activation activity of HIF-1α (Fig. 3c) and promoted the transcription of its target genes (Fig. 3d).

### Celastrol-mediated HIF-1α accumulation stimulates BNIP3 expression and induces autophagy

**Figure 4:**
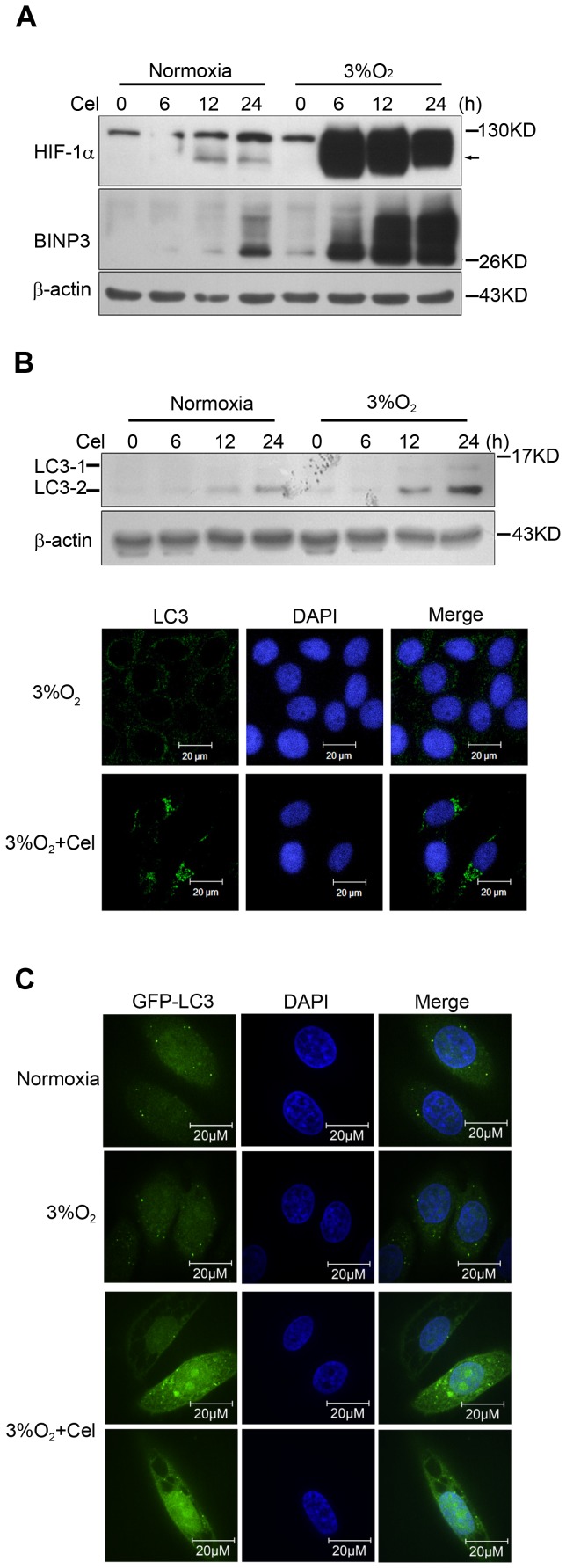
Celastrol-mediated HIF-1α accumulation stimulates BNIP3 expression and induces mitochondrial autophagy. 4a. Celastrol time-dependently enhanced the expression of HIF-1α and BNIP3. HepG2 cells were treated with 4 µM Celastrol in normoxia or hypoxia for the indicated times. The protein levels were determined by western blot analysis. 4b. Celastrol enhances LC3-II expression and the formation of LC-3 aggregates. HepG2 cells were treated with 4 µM Celastrol under normoxia or hypoxia for the indicated times. The LC3I/II protein levels were determined by western blot analysis (upper), and the LC3 aggregates were stained by indirect immunofluorescence and observed by confocal microscopy. 4c. Celastrol induced autophagy. HepG2 cells were transiently transfected with GFP-LC3, and 24 h later, the cells were treated with 4 µM Celastrol for another 24 h. GFP fluorescence was then observed by confocal microscopy.

Previously, many studies have reported that HIF-1α induces the expression of Bcl2/adenovirus E1B 19 kD-interacting protein 3 (BNIP3), which triggers selective mitochondrial autophagy [23], [24]. Therefore, we analyzed the influence of Celastrol-induced HIF-1α accumulation on BNIP3 expression. Western blotting demonstrated that Celastrol time-dependently enhanced the protein level of BNIP3, which was coincident with enhanced HIF-1α (Fig. 4a). The microtubule-associated protein 1 light chain 3 (LC3) has been used as a marker for autophagy because, upon induction of autophagy, some LC3-I is converted into LC3-II. We detected the expression of LC3 by indirect immunofluorescence staining and western blotting, and the results showed that Celastrol time-dependently increased LC3-II expression, and followed Celastrol exposure, the formation of LC3 aggregates became significant (Fig. 4b). To further confirm this change, HepG2 cells were transfected with the GFP-LC3 plasmid, and the cells were treated with or without Celastrol for 24 h and then observed using a confocal microscope. As Fig. 4c shows, in control cells, GFP-LC3 was evenly distributed throughout the entire cytoplasm; however, following Celastrol treatment, the formation of GFP-LC3 aggregates also became significant, indicating the formation of LC3-II, and more vacuole-like structures appeared, indicating that Celastrol induced autophagy.

### Knockdown of HIF-1α or BNIP3 did not influence Celastrol-induced cell apoptosis under hypoxia

**Figure 5:**
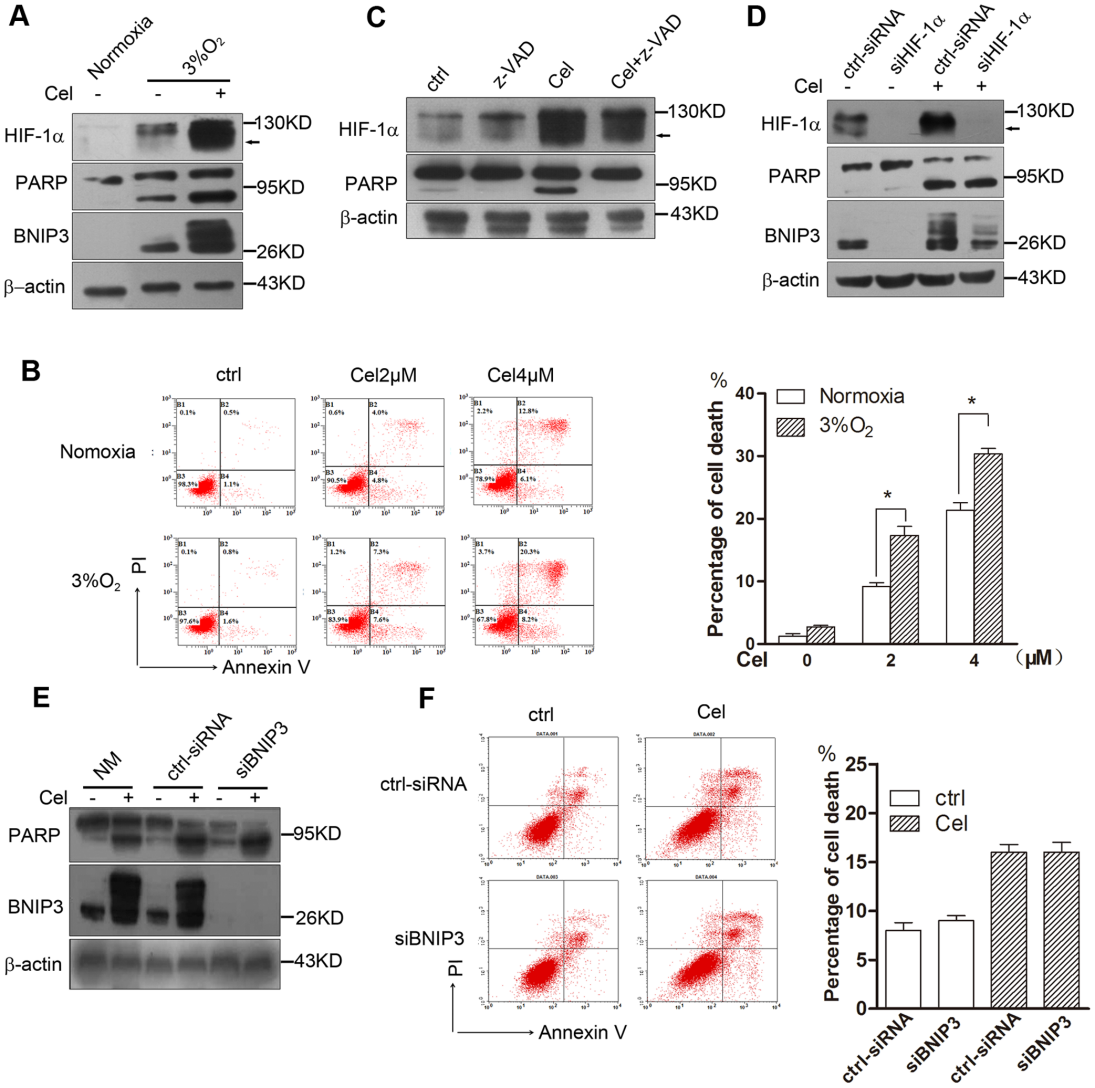
Knockdown of HIF-1α or BNIP3 did not influence Celastrol-induced cell apoptosis under hypoxia. 5a. Celastrol enhanced the HIF-1α and BNIP3 expression that was accompanied by increased PARP cleavage under hypoxia. HepG2 cells were cultured under normoxia or hypoxia with or without 4 µM Celastrol for 24 h. Western blotting was used to detect protein expression. 5b. The cytotoxicity of Celastrol was enhanced under hypoxia. HepG2 cells were cultured under normoxia or hypoxia with or without 2–4 µM Celastrol for 24 h. The cells were then stained with Annexin-V/PI and analyzed by flow cytometry. The data are presented as the mean values obtained from three independent experiments. 5c. Z-VAD blocked Celastrol-induced PARP cleavage but did not affect HIF-1α accumulation. HepG2 cells were pre-treated with 10 µM zVAD for 1 h then exposed to 4 µM Celastrol for another 6 h. The proteins were detected by western blot analysis. 5d. Knockdown of HIF-1α did not affect Celastrol-induced PARP cleavage. HepG2 cells were transfected with a non-silencing siRNA or HIF-1α siRNA for 24 h, and the cells were then treated with 4 µM Celastrol for 24 h. The proteins were detected by western blot analysis. 5e, 5f. Knockdown of BNIP3 did not affect Celastrol-induced cell death. HepG2 cells were transfected with non-silencing siRNA or BNIP3 siRNA for 24 h, and the cells were then treated with 4 µM Celastrol for 24 h. Cell death was measured by Annexin-V/PI staining and flow cytometry, and the proteins were detected by western blotting.

Previous studies have reported that hypoxia-mediated expression of BNIP3 has the potential to protect cells [25] [26]; however, we observed that, under hypoxia, although Celastrol significantly increased the expression of HIF-1α and BNIP3, it still enhanced the expression of the cleaved-PARP protein (Fig. 5a). Annexin V/PI staining and flow cytometry analysis also showed that the cytotoxicity of Celastrol was enhanced under hypoxia (Fig. 5b). Using the caspase inhibitor Z-VAD to pretreat HepG2 cells completely blocked Celastrol-induced PARP cleavage, but it did not affect HIF-1α accumulation (Fig. 5c). To confirm whether the Celastrol-induced accumulation of HIF-1α or BNIP3 could affect cell death, we evaluated the effect of knockdown of HIF-1α or BNIP3 on Celastrol-induced cell death using siRNAs. The result showed that knocking down HIF-1α expression completely inhibited BNIP3 expression, but it did not affect PARP cleavage induced by 4 µM Celastrol (Fig. 5d). Similarly, knockdown of BNIP3 did not affect Celastrol-induced PARP cleavage or cell death (Figs. 5e, 5f). These data indicated that the accumulation of HIF-1α and HIF-1α-mediated BNIP3 protein did not involve Celastrol-induced cell death.

### Celastrol kills cancer cells via ROS-mediated JNK activation

**Figure 6:**
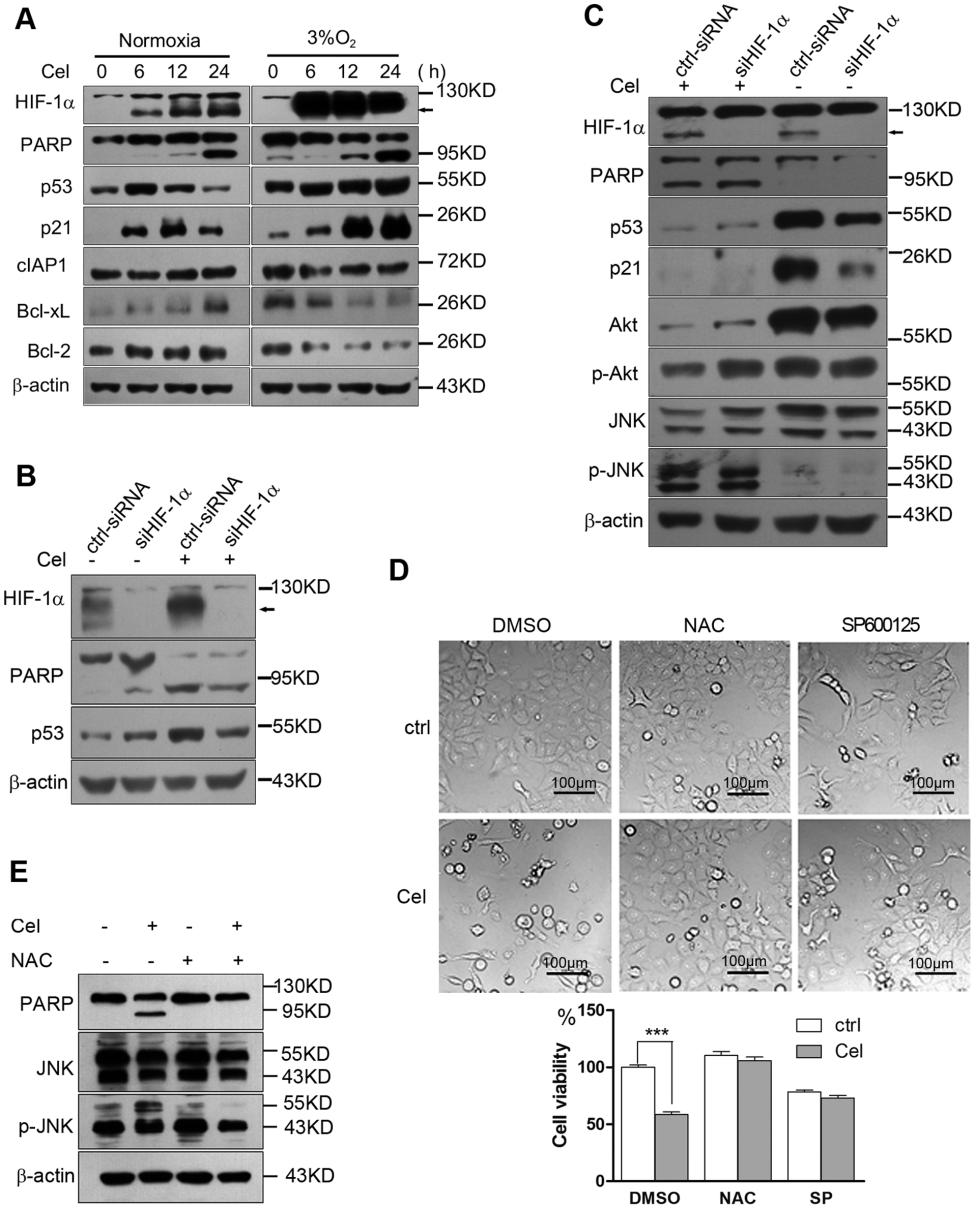
Celastrol kills HepG2 cells via ROS-mediated JNK activation. 6a. Celastrol transiently activates p53 under normoxia but persistently activates p53 under hypoxia. HepG2 cells were cultured in normoxia or hypoxia with or without 4 µM Celastrol for the indicated times. The proteins were detected by western blotting. 6b. The effect of HIF1α knockdown on p53 expression under hypoxia. HepG2 cells were transfected with control or HIF1α siRNA for 24 h and then treated with or without 4 µM Celastrol under hypoxia for 24 h. The proteins were detected by western blotting. 6c. The effect of HIF1α knockdown on Celastrol-induced p53 expression and Akt and JNK activation under normoxia. HepG2 cells were transfected with control or HIF1α siRNA for 24 h and then treated with or without 4 µM Celastrol under normoxia for 24 h. The proteins were detected by western blotting. 6d, 6e. Suppression of ROS-induced JNK activity could prevent Celastrol-mediated cell death. HepG2 cells were pretreated with either the ROS scavenger 5 mM NAC or the JNK kinase inhibitor 40 µM SP600125 for 1 h and then treated with or without 4 µM Celastrol under normoxia for 24 h. Cell death was examined by microscopy (200×), as based on morphological changes, and quantified using the MTT assay. The proteins were detected by western blotting.

**Figure 7:**
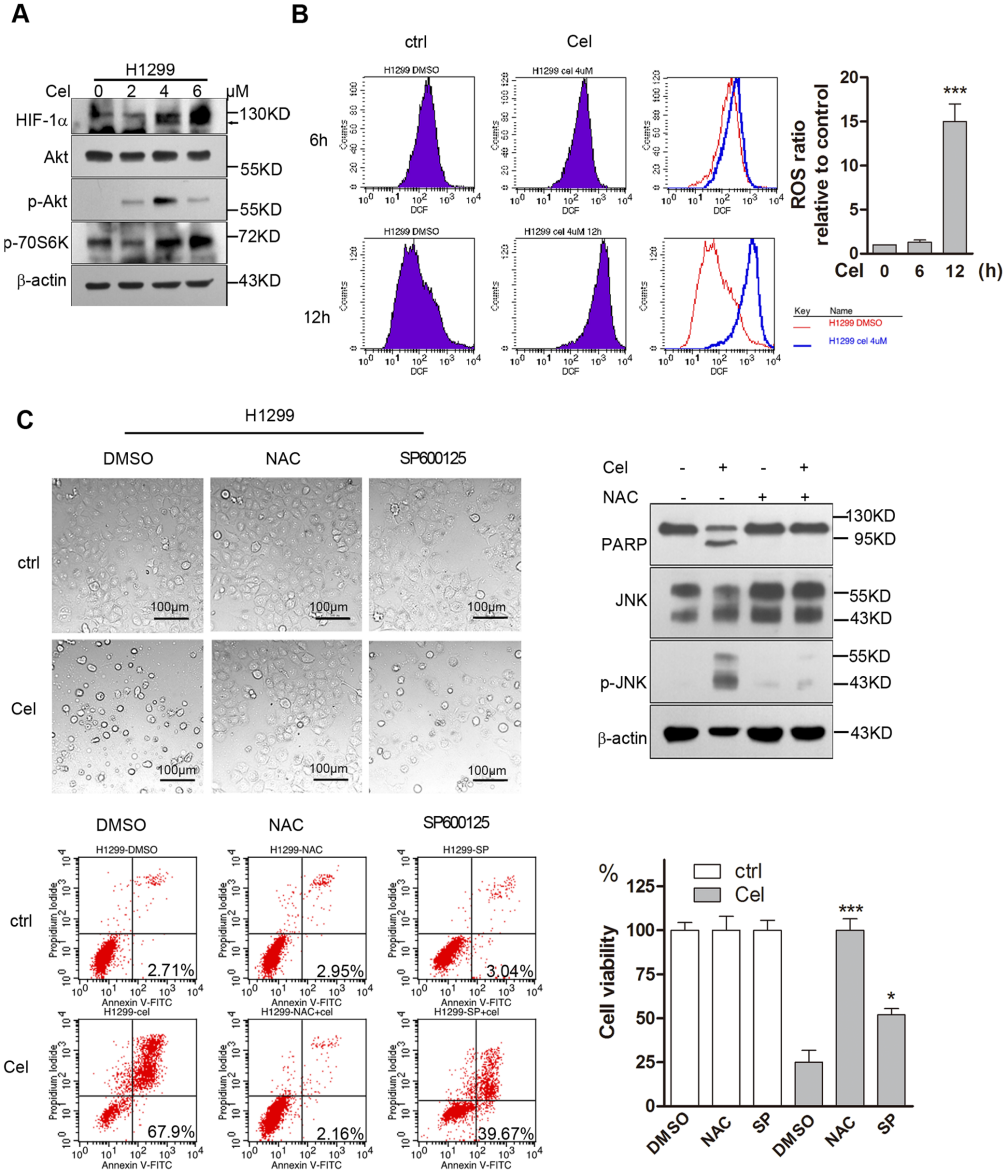
Celastrol kills p53-null H1299 cells via ROS-mediated JNK activation. 7a. Celastrol enhances HIF-1α expression and induces Akt activation in H1299 cells. H1299 cells were treated with the indicated doses of Celastrol for 6 h, and the proteins were detected by western blotting. 7b. Celastrol time-dependently induces ROS accumulation in H1299 cells. H1299 cells were treated with 4 µM Celastrol for the indicated times. The levels of ROS were measured by DCFH-DA staining and subsequently assayed by flow cytometry. 7c. Suppression of ROS-induced JNK activity could prevent Celastrol-mediated H1299 cell death. H1299 cells were pretreated with either the ROS scavenger 5 mM NAC or the JNK kinase inhibitor 40 µM SP600125 for 1 h and then treated with or without 4 µM Celastrol under normoxia for 24 h. Cell death was examined by microscopy (200×) and measured by Annexin-V/PI staining and flow cytometry. The proteins were detected by western blotting.

To further investigate the possible mechanism by which Celastrol kills cancer cells, we detected the expression of several proteins in HepG2 cells after 4 µM Celastrol exposure for the indicated times. We found that Celastrol induced the activation of p53, but the dynamic changes in p53 expression were different under normoxia and hypoxia. Under normoxia, Celastrol transiently enhanced the expression of the p53 protein at 6 h, but the p53 protein level declined at 24 h (Fig. 6a). In contrast, under hypoxia, Celastrol persistently enhanced the expression of p53 and its target protein p21 (Fig. 6a). As a key mediator of cellular stress responses, HIF1α has been reported to either stimulate or suppress p53 depending on the oxygen conditions [27]. Our results showed that siRNA knockdown of HIF1α under hypoxia could enhance p53 expression; however, Celastrol-induced p53 activation was weakened, indicating that Celastrol-induced p53 activation is at least partially dependent on HIF-1(Fig. 6b). In contrast, under normoxia, HIF1α knockdown decreased the intrinsic expression of p53 and p21 (Fig. 6c), and similar to the results shown in Fig. 6a, treating HepG2 cells with Celastrol under normoxia for 24 h obviously suppressed p53 activation, reduced p21 expression and, at the same time, induced PARP cleavage (Fig. 6c). These data do not support that the transient or lasting activation of p53 that is induced by Celastrol in normoxia or hypoxia involves cell killing. In addition to p53, we observed that Celastrol induced remarkable JNK activation, which was not affected by HIF-1α knockdown (Fig. 6c). More importantly, unlike Celastrol-induced Akt activation at 6 h (Fig. 2e, 2f, 2 h), treating cells with Celastrol for 24 h induced a remarkable depletion of the total Akt protein and reduced phosphor-Akt (Fig. 6c). HIF-1α knockdown lessened the inhibition of Akt activation, but it did not save the cells from Celastrol-induced apoptosis, as the cells showed the same amount of PARP cleavage as the control (Fig. 6c). Next, we used the ROS scavenger NAC or the JNK kinase inhibitor SP600125 to treat HepG2 cells incubated with or without 4 µM Celastrol for 24 h and observed their effects on Celastrol-induced cell death. The results showed that NAC could completely block the cytotoxicity of Celastrol, whereas SP600125 inhibited cell proliferation and rescued the cells from cell death (Fig. 6d). Western blotting also showed that NAC could completely inhibit Celastrol-induced JNK activation and block PARP cleavage (Fig. 6e), indicating that Celastrol-induced ROS accumulation and JNK activation were the important mechanisms for cell killing. To further support this conclusion, we repeated these experiments in H1299 cells, which are p53-null cells. Like HepG2 cells, H1299 cells treated with 2–6 µM Celastrol for 6 h could activate Akt and induce HIF-1α protein accumulation (Fig. 7a). Celastrol also time-dependently induced ROS production (Fig. 7b), and NAC blocked Celastrol-induced JNK activation and rescued cell death (Fig. 7c).

### Celastrol could stimulate HIF-1α accumulation with a dose below its cytotoxic threshold

**Figure 8:**
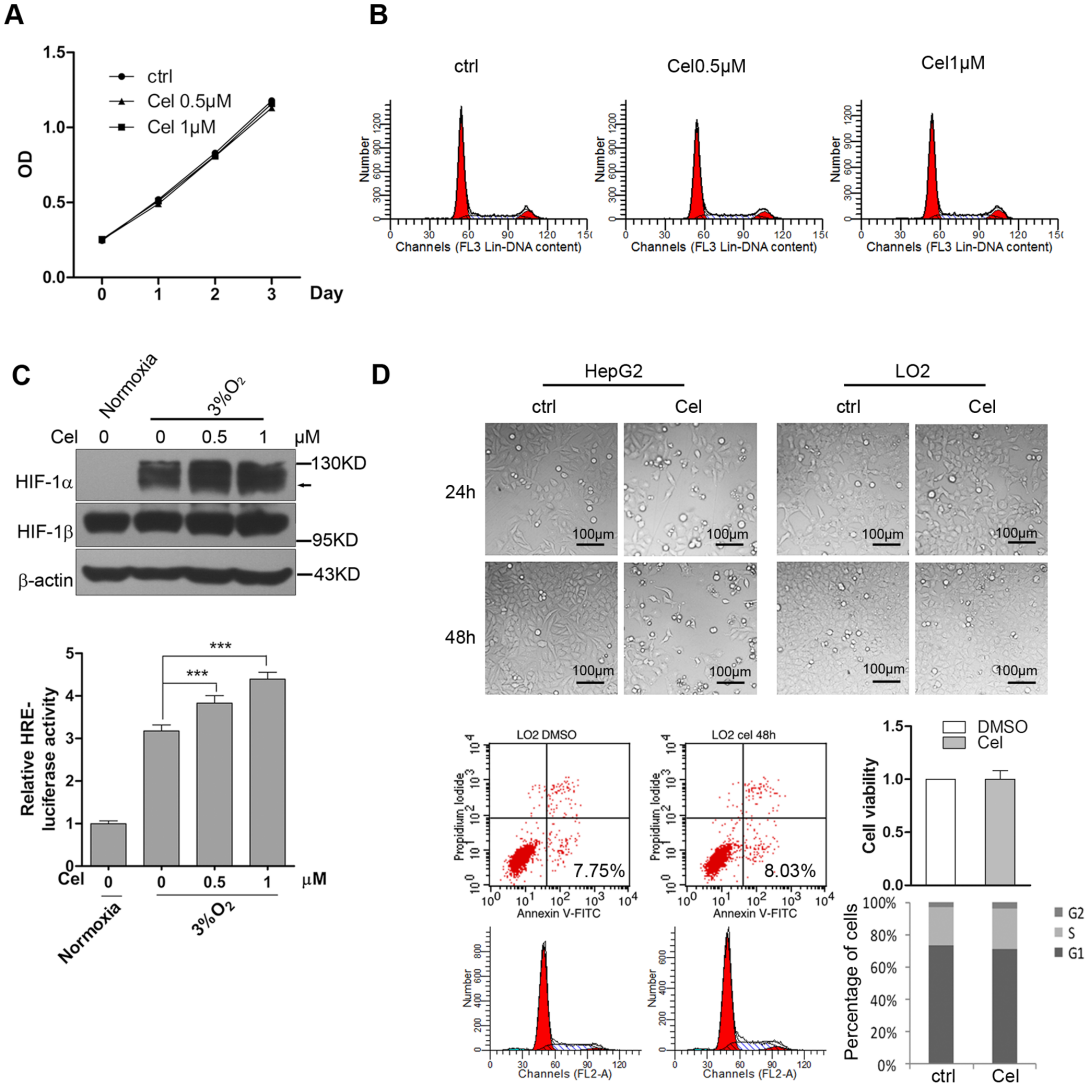
Celastrol could stimulate HIF-1α accumulation with a dose below its cytotoxic threshold. 8a. Low dose of Celastrol stimulated HIF-1α accumulation. HepG2 cells were treated with 0.5–1 µM Celastrol for 24 h in normoxia and hypoxia, and the viability of the cells was then detected by the MTT assay. 8b. 0.5–1 µM Celastrol did not arrest the cell cycle. HepG2 cells were treated with 0.5–1 µM Celastrol for 12 h in normoxia, and cell cycle analysis was performed using PI staining and flow cytometry. 8c. The effects of a low dose of Celastrol on HIF-1α protein expression and its transcriptional activation activity. HepG2 cells were treated with 0.5–1 µM Celastrol for 12 h in normoxia, and protein expression was determined using western blotting (upper). The transcriptional activation activity of HIF-1 was determined by transient transfection of HepG2 cells with HRE-luciferase reporter plasmids for 24 h followed by Celastrol exposure under normoxia for another 12 h. Then, HRE activity was analyzed using the luciferase assay (lower). The values are presented as the means ± SD of three independent experiments. 8d. The effect of Celastrol on LO2 normal liver cells. LO2 cells and HepG2 cells were treated with 4 µM Celastrol for 24–48 h, and the cytotoxicity of Celastrol was observed by microscopy (200×). Cell death and the cell cycle progression of the LO2 cells were measured by flow cytometry.

To investigate whether HIF-1α signal activation by Celastrol is correlated with cytotoxicity, we treated HepG2 cells with 0.5–1 µM Celastrol for 0–72 h and detected the cell-growth rate using the MTT assay. The result showed that Celastrol did not inhibit cell proliferation with a dose of less than 1 µM (Fig. 8a), and this conclusion was further confirmed by flow cytometric analysis of the cell cycle (Fig. 8b). Subsequently, we treated the cells with the indicated low dose of Celastrol and detected the accumulation of the HIF-1α protein and its transcription activity under hypoxia using the methods described above. The results showed that Celastrol could still enhance HIF-1α expression and active HIF-1 signaling at this dose without causing cytotoxicity (Fig. 8c). In addition, we observed the cytotoxicity of Celastrol in a normal liver cell line, LO2. It is interesting to note that, unlike in cancer cells, treating the cells with 4 µM Celastrol for as long as 48 h did not arrest the cell cycle or induce cell death (Fig. 8d).

## Discussion

In this study, we found, for the first time, that Celastrol could induce accumulation of the HIF-1α protein in an oxygen-independent manner, and the accumulation of HIF-1α increased the expression of BNIP3, which induced autophagy. A previous study showed that treating HepG2 cells with Celastrol for 16 h decreased the HIF-1α mRNA level under normoxia and hypoxia and inhibited hypoxia-induced accumulation of the HIF-1α protein in the nuclei of HepG2 cells [15]; however, in our study, exposure of HepG2 cells for a shorter amount of time yielded the opposite result. In our system, treating cells with Celastrol for 6 h did not influence HIF-1α transcription, as its mRNA level did not change after Celastrol treatment under normoxia or hypoxia. Furthermore, Celastrol induced HIF-1α protein accumulation, which then entered the nucleus and promoted HIF-1 target-gene transcription. The difference in exposure time could explain these discrepant results. The effect of Celastrol in enhancing HIF-1α expression was not specific to HepG2 cells because this effect could be observed in other cell lines, including MCF-7, HeLa, PC-3 and H1299 cells. Secondly, we demonstrated that the mechanism by which Celastrol induces the accumulation of the HIF-1α protein is inducing ROS and activating Akt/p70S6K signaling to promote HIF-1α translation.

Although the protein level of HIF-1α is normally regulated by oxygen-dependent, pVHL-mediated ubiquitination and degradation [28], other molecules, such as p53 and Hsp90, have also been identified to regulate the stability of HIF-1α [18], [29]. Celastrol has been reported to be a potent proteasome [19] and Hsp90 inhibitor [30], [31]; however, these two functions should theoretically have opposing effects on the stability of HIF-1α. Therefore, the mechanism by which Celastrol affects HIF-1 signaling is unclear. In this study, we found that Celastrol did not enhance HIF-1α ubiquitination, as did the proteasome inhibitor MG132. Furthermore, as an Hsp90 inhibitor, Celastrol depleted other Hsp90 client proteins, such as Raf-1 and Akt, but it enhanced HIF-1α expression, though HIF-1α is also a client of Hsp90 [32]. Moreover, the enhancement of HIF-1α protein levels caused by Celastrol was independent of p53 and pVHL-mediated hydroxylation, but it required new protein synthesis, which indicated that the regulation of HIF-1α by Celastrol did not involve inhibition of HIF-1α degradation.

In addition to oxygen-dependent regulation, it is known that HIF-1 is activated or influenced through oxygen-independent mechanisms via the PI3K/AKT/mTOR pathways [22], [33]–[35]. The activation of Akt was reported to augment HIF-1α expression by increasing its translation under normoxic and hypoxic conditions [36], and the Akt/mTOR-dependent translation of HIF-1α was reported to play a critical role in the post-irradiation up-regulation of intratumoral HIF-1 activity [22]. Although a previous study showed that mitochondrial ROS produced under hypoxia played an important role in stabilization of the HIF-1α protein by inhibiting prolyl hydroxylase enzymes [37], further studies revealed that mitochondrial ROS-upregulated HIF-1α expression is dependent upon PI3K/AKT activity [38]–[40]. Previously, we reported that Celastrol targets mitochondrial respiratory chain complex I to induce ROS-dependent cytotoxicity in tumor cells [41]. In this study, we showed that the enhancement of HIF-1α expression by Celastrol was ROS- and PI3K/AKT-dependent. This finding supported the hypothesis that the promotion of HIF-1α expression by Celastrol is correlated with ROS-initiated AKT activation, which enhances HIF-1α translation. As an important transcriptional factor, the accumulation of HIF-1α may affect multiple signaling pathways and regulate various biological functions, such as inducing autophagy [42], [43], promoting tumor cell invasion and metastasis [44]–[46] and protecting cells of the brain, liver, kidney and heart from cellular oxidative stress and ischemia/reperfusion-induced injury [47]–[49]. Although Celastrol induced the transient accumulation of HIF-1α and VEGF, whether it can promote tumor angiogenesis and metastasis is a remaining question. Previous studies have shown that Celastrol could suppress tumor invasion and metastasis [14], [50]–[52]; however, when considering that radiation-induced HIF-1α activation plays a crucial role in triggering tumor radioresistance [53], [54], the effects of Celastrol on tumor angiogenesis and vasculogenesis may warrant further investigation.

BNIP3 is an atypical BH3-only family member that has been implicated in the pathogenesis of cancer and heart disease. Previous studies have reported that mitochondrial autophagy induced by hypoxia requires the HIF-1-dependent expression of BNIP3, which plays a protective role by disrupting the Bcl-2-Beclin1 complex without inducing cell death in tumor cells [42], [43]. Similarly, in heart muscle, the expression of BNIP3 that is regulated by hypoxia is associated with decreased myocardial function via the induction of autophagy [26], [55]. Previous studies have found that Celastrol induces autophagy, but the mechanism is unclear [56], [57]. Our data showed that the activation of HIF-1/BNIP3 signaling is an important mechanism for Celastrol to induce autophagy. Because the dose required for Celastrol to induce accumulation of the HIF-1α protein and enhance HIF-1α transcriptional activation is below its cytotoxic threshold and because normal cells are very resistant to Celastrol, using a low dose of Celastrol to activate the HIF-1-mediated autophagic pathway could be a good strategy for utilizing the neuroprotective effects of Celastrol while avoiding its cytotoxicity. A recent study has demonstrated that Celastrol protects human neuroblastoma SH-SY5Y cells from rotenone-induced injuries only through the induction of autophagy [56].

**Figure 9:**
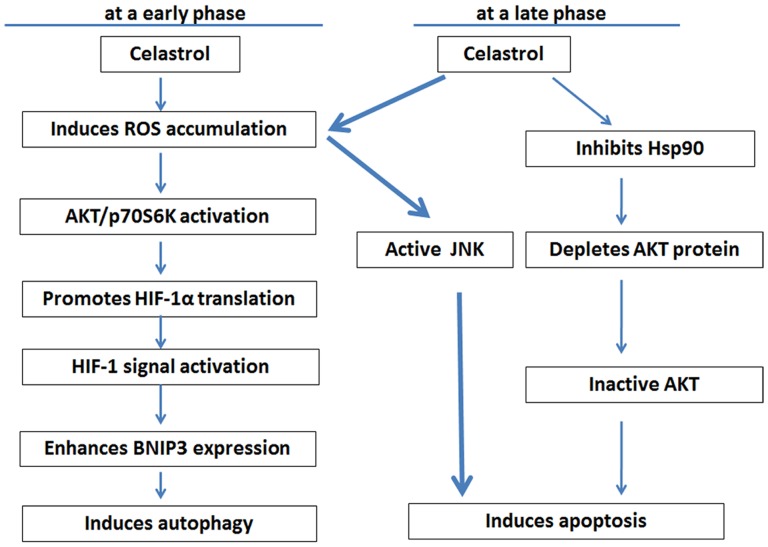
The mechanism by which Celastrol induces autophagy and apoptosis. In our system, we showed that Celastrol could induce ROS-mediated autophagy and apoptosis. Celastrol-induced autophagy occurred at an early phase and was initiated by ROS to stimulate AKT/p70S6K signal activation, which promotes HIF-1α translation and BNIP3 expression, leading to autophagy. Apoptosis occurred at a late phase, and the main mechanism by which Celastrol killed HepG2 and H1299 cells was through the ROS-mediated activation of JNK and inhibition of Hsp90 chaperone function, which led to Akt protein depletion and a decrease in Akt activity, finally resulting in the induction of apoptosis.

Previous studies have demonstrated that Celastrol exerts its anticancer effects by suppressing Akt activation in tumor cells [14], [58]. In this study, we observed that Celastrol induced Akt activation and enhanced HIF-1α expression, which seems contradictory to the previous conclusion. However, it is worth noting that the effect of Celastrol on Akt activation was transient and occurred early (exposure for 2–6 h) and that Celastrol continually inhibited Hsp90 chaperone function; therefore, the Akt protein was remarkably depleted, and the activity of Akt was finally inhibited (Fig. 6c), which is consistent with previous studies. Because knockdown of HIF-1α partially relieved Celastrol-induced Akt suppression but failed to reduce cell death, we believe that suppression of Akt activation only partially contributed to Celastrol-induced HepG2 cell death. ROS-mediated JNK activation has been reported to lead to cancer cell apoptosis [58]. In our experiments in both HepG2 and H1299 cells, Celastrol induced remarkable ROS accumulation, and ROS scavenging suppressed ROS-induced JNK activation and prevented cell death, which supported that mitochondrial targeting and ROS induction are the important mechanisms by which Celastrol kills cancer cells, as previously reported [41]. Based on these discoveries, we have summarized the main mechanism for the induction of autophagy or apoptosis by Celastrol in a schematic diagram (Fig. 9).

## References

[1] O'Rourke JF, Dachs GU, Gleadle JM, Maxwell PH, Pugh CW, et al (1997) Hypoxia response elements. Oncol Res 9: 327–332. 9406238

[2] D'Angelo G, Duplan E, Boyer N, Vigne P, Frelin C (2003) Hypoxia up-regulates prolyl hydroxylase activity: a feedback mechanism that limits HIF-1 responses during reoxygenation. J Biol Chem 278: 38183–38187. 12876291 10.1074/jbc.M302244200

[3] Calixto JB, Campos MM, Otuki MF, Santos ARS (2004) Anti-inflammatory compounds of plant origin. Part II. Modulation of pro-inflammatory cytokines, chemokines and adhesion molecules. Planta Med 70: 93–103. 14994184 10.1055/s-2004-815483

[4] Venkatesha SH, Yu H, Rajaiah R, Tong L, Moudgil KD (2011) Celastrus-derived celastrol suppresses autoimmune arthritis by modulating antigen-induced cellular and humoral effector responses. J Biol Chem 286: 15138–15146. 21402700 10.1074/jbc.M111.226365 PMC3083183

[5] Faust K, Gehrke S, Yang Y, Yang L, Beal MF, et al (2009) Neuroprotective effects of compounds with antioxidant and anti-inflammatory properties in a Drosophila model of Parkinson's disease. BMC Neurosci 10: 109. 19723328 10.1186/1471-2202-10-109 PMC3152779

[6] Ge P, Ji X, Ding Y, Wang X, Fu S, et al (2010) Celastrol causes apoptosis and cell cycle arrest in rat glioma cells. Neurol Res 32: 94–100. 19909582 10.1179/016164109X12518779082273

[7] Dai Y, Desano J, Tang W, Meng X, Meng Y, et al (2010) Natural proteasome inhibitor celastrol suppresses androgen-independent prostate cancer progression by modulating apoptotic proteins and NF-kappaB. PLoS One 5: e14153. 21170316 10.1371/journal.pone.0014153 PMC3000808

[8] Mou H, Zheng Y, Zhao P, Bao H, Fang W, et al (2011) Celastrol induces apoptosis in non-small-cell lung cancer A549 cells through activation of mitochondria- and Fas/FasL-mediated pathways. Toxicol In Vitro 25: 1027–1032. 21466843 10.1016/j.tiv.2011.03.023

[9] Sha M, Ye J, Zhang LX, Luan ZY, Chen YB (2013) Celastrol induces apoptosis of gastric cancer cells by miR-146a inhibition of NF-kappaB activity. Cancer Cell Int 13: 50. 23706078 10.1186/1475-2867-13-50 PMC3672015

[10] Sethi G, Ahn KS, Pandey MK, Aggarwal BB (2007) Celastrol, a novel triterpene, potentiates TNF-induced apoptosis and suppresses invasion of tumor cells by inhibiting NF-kappaB-regulated gene products and TAK1-mediated NF-kappaB activation. Blood 109: 2727–2735. 17110449 10.1182/blood-2006-10-050807

[11] Dai Y, DeSano JT, Meng Y, Ji Q, Ljungman M, et al (2009) Celastrol potentiates radiotherapy by impairment of DNA damage processing in human prostate cancer. Int J Radiat Oncol Biol Phys 74: 1217–1225. 19545787 10.1016/j.ijrobp.2009.03.057 PMC2745184

[12] Jo H, Loison F, Hattori H, Silberstein LE, Yu H, et al (2010) Natural product Celastrol destabilizes tubulin heterodimer and facilitates mitotic cell death triggered by microtubule-targeting anti-cancer drugs. PLoS One 5: e10318. 20428237 10.1371/journal.pone.0010318 PMC2859055

[13] Zhu H, Ding WJ, Wu R, Weng QJ, Lou JS, et al (2010) Synergistic anti-cancer activity by the combination of TRAIL/APO-2L and celastrol. Cancer Invest 28: 23–32. 19916747 10.3109/07357900903095664

[14] Pang X, Yi Z, Zhang J, Lu B, Sung B, et al (2010) Celastrol suppresses angiogenesis-mediated tumor growth through inhibition of AKT/mammalian target of rapamycin pathway. Cancer Res 70: 1951–1959. 20160026 10.1158/0008-5472.CAN-09-3201 PMC2854134

[15] Huang L, Zhang Z, Zhang S, Ren J, Zhang R, et al (2011) Inhibitory action of Celastrol on hypoxia-mediated angiogenesis and metastasis via the HIF-1alpha pathway. Int J Mol Med 27: 407–415. 21249310 10.3892/ijmm.2011.600

[16] Liu XH, Kirschenbaum A, Yao S, Stearns ME, Holland JF, et al (1999) Upregulation of vascular endothelial growth factor by cobalt chloride-simulated hypoxia is mediated by persistent induction of cyclooxygenase-2 in a metastatic human prostate cancer cell line. Clin Exp Metastasis 17: 687–694. 10919714 10.1023/a:1006728119549

[17] Pfaffl MW (2001) A new mathematical model for relative quantification in real-time RT-PCR. Nucleic Acids Res 29: e45. 11328886 10.1093/nar/29.9.e45 PMC55695

[18] Kaluzova M, Kaluz S, Lerman MI, Stanbridge EJ (2004) DNA damage is a prerequisite for p53-mediated proteasomal degradation of HIF-1alpha in hypoxic cells and downregulation of the hypoxia marker carbonic anhydrase IX. Mol Cell Biol 24: 5757–5766. 15199132 10.1128/MCB.24.13.5757-5766.2004 PMC480909

[19] Yang H, Chen D, Cui QC, Yuan X, Dou QP (2006) Celastrol, a triterpene extracted from the Chinese “Thunder of God Vine,” is a potent proteasome inhibitor and suppresses human prostate cancer growth in nude mice. Cancer Res 66: 4758–4765. 16651429 10.1158/0008-5472.CAN-05-4529

[20] Walcott SE, Heikkila JJ (2010) Celastrol can inhibit proteasome activity and upregulate the expression of heat shock protein genes, hsp30 and hsp70, in Xenopus laevis A6 cells. Comp Biochem Physiol A Mol Integr Physiol 156: 285–293. 20188206 10.1016/j.cbpa.2010.02.015

[21] Schnitzer SE, Schmid T, Zhou J, Eisenbrand G, Brune B (2005) Inhibition of GSK3beta by indirubins restores HIF-1alpha accumulation under prolonged periods of hypoxia/anoxia. FEBS Lett 579: 529–533. 15642371 10.1016/j.febslet.2004.12.023

[22] Harada H, Itasaka S, Kizaka-Kondoh S, Shibuya K, Morinibu A, et al (2009) The Akt/mTOR Pathway Assures the Synthesis of HIF-1 alpha Protein in a Glucose- and Reoxygenation-dependent Manner in Irradiated Tumors. Journal of Biological Chemistry 284: 5332–5342. 19098000 10.1074/jbc.M806653200

[23] Zhang H, Bosch-Marce M, Shimoda LA, Tan YS, Baek JH, et al (2008) Mitochondrial autophagy is an HIF-1-dependent adaptive metabolic response to hypoxia. J Biol Chem 283: 10892–10903. 18281291 10.1074/jbc.M800102200 PMC2447655

[24] Band M, Joel A, Hernandez A, Avivi A (2009) Hypoxia-induced BNIP3 expression and mitophagy: in vivo comparison of the rat and the hypoxia-tolerant mole rat, Spalax ehrenbergi. FASEB J 23: 2327–2335. 19255257 10.1096/fj.08-122978

[25] Tracy K, Macleod KF (2007) Regulation of mitochondrial integrity, autophagy and cell survival by BNIP3. Autophagy 3: 616–619. 17786027 10.4161/auto.4892 PMC2989881

[26] Hamacher-Brady A, Brady NR, Gottlieb RA, Gustafsson AB (2006) Autophagy as a protective response to Bnip3-mediated apoptotic signaling in the heart. Autophagy 2: 307–309. 16874059 10.4161/auto.2947

[27] Obacz J, Pastorekova S, Vojtesek B, Hrstka R (2013) Cross-talk between HIF and p53 as mediators of molecular responses to physiological and genotoxic stresses. Mol Cancer 12: 93. 23945296 10.1186/1476-4598-12-93 PMC3844392

[28] Wei W, Yu XD (2007) Hypoxia-inducible factors: crosstalk between their protein stability and protein degradation. Cancer Lett 257: 145–156. 17920193 10.1016/j.canlet.2007.08.009

[29] Minet E, Mottet D, Michel G, Roland I, Raes M, et al (1999) Hypoxia-induced activation of HIF-1, role of HIF-1 alpha-Hsp90 interaction. Febs Letters 460: 251–256. 10544245 10.1016/s0014-5793(99)01359-9

[30] Zhang T, Li Y, Yu Y, Zou P, Jiang Y, et al (2009) Characterization of celastrol to inhibit hsp90 and cdc37 interaction. J Biol Chem 284: 35381–35389. 19858214 10.1074/jbc.M109.051532 PMC2790967

[31] Chadli A, Felts SJ, Wang Q, Sullivan WP, Botuyan MV, et al (2010) Celastrol inhibits Hsp90 chaperoning of steroid receptors by inducing fibrillization of the Co-chaperone p23. J Biol Chem 285: 4224–4231. 19996313 10.1074/jbc.M109.081018 PMC2823561

[32] Liu YV, Baek JH, Zhang H, Diez R, Cole RN, et al (2007) RACK1 competes with HSP90 for binding to HIF-1 alpha and is required for O-2-independent and HSP90 inhibitor-induced degradation of HIF-1 alpha. Molecular Cell 25: 207–217. 17244529 10.1016/j.molcel.2007.01.001 PMC2563152

[33] Xie SR, Wang Y, Liu CW, Luo K, Cai YQ (2012) Liquiritigenin Inhibits Serum-induced HIF-1a and VEGF Expression via the AKT/mTOR-p70S6K Signalling Pathway in HeLa Cells. Phytotherapy Research 26: 1133–1141. 22170854 10.1002/ptr.3696

[34] Majumder PK, Febbo PG, Bikoff R, Berger R, Xue Q, et al (2004) mTOR inhibition reverses Akt-dependent prostate intraepithelial neoplasia through regulation of apoptotic and HIF-1-dependent pathways. Nature Medicine 10: 594–601. 10.1038/nm1052 15156201

[35] Agani F, Jiang BH (2013) Oxygen-independent Regulation of HIF-1: Novel Involvement of PI3K/AKT/mTOR Pathway in Cancer. Curr Cancer Drug Targets 13: 245–251. 23297826 10.2174/1568009611313030003

[36] Pore N, Jiang ZB, Shu HK, Bernhard E, Kao GD, et al (2006) Akt1 activation can augment hypoxia-inducible factor-1 alpha expression by increasing protein translation through a mammalian target of rapamycin-independent pathway. Molecular Cancer Research 4: 471–479. 16849522 10.1158/1541-7786.MCR-05-0234

[37] Chandel NS, McClintock DS, Feliciano CE, Wood TM, Melendez JA, et al (2000) Reactive oxygen species generated at mitochondrial complex III stabilize hypoxia-inducible factor-1 alpha during hypoxia - A mechanism of O-2 sensing. Journal of Biological Chemistry 275: 25130–25138. 10833514 10.1074/jbc.M001914200

[38] Gao N, Ding M, Zheng JZ, Zhang Z, Leonard SS, et al (2002) Vanadate-induced expression of hypoxia-inducible factor 1 alpha and vascular endothelial growth factor through phosphatidylinositol 3-kinase/Akt pathway and reactive oxygen species. Journal of Biological Chemistry 277: 31963–31971. 12070140 10.1074/jbc.M200082200

[39] Koshikawa N, Hayashi JI, Nakagawara A, Takenaga K (2009) Reactive Oxygen Species-generating Mitochondrial DNA Mutation Up-regulates Hypoxia-inducible Factor-1 alpha Gene Transcription via Phosphatidylinositol 3-Kinase-Akt/Protein Kinase C/Histone Deacetylase Pathway. Journal of Biological Chemistry 284: 33185–33194. 19801684 10.1074/jbc.M109.054221 PMC2785161

[40] Guo LL, Li L, Wang WQ, Pan ZH, Zhou QH, et al (2012) Mitochondrial reactive oxygen species mediates nicotine-induced hypoxia-inducible factor-1 alpha expression in human non-small cell lung cancer cells. Biochimica Et Biophysica Acta-Molecular Basis of Disease 1822: 852–861. 10.1016/j.bbadis.2012.02.004 22349311

[41] Chen G, Zhang X, Zhao M, Wang Y, Cheng X, et al (2011) Celastrol targets mitochondrial respiratory chain complex I to induce reactive oxygen species-dependent cytotoxicity in tumor cells. BMC Cancer 11: 170. 21569548 10.1186/1471-2407-11-170 PMC3112161

[42] Mazure NM, Pouyssegur J (2009) Atypical BH3-domains of BNIP3 and BNIP3L lead to autophagy in hypoxia. Autophagy 5: 868–869. 19587545 10.4161/auto.9042

[43] Bellot G, Garcia-Medina R, Gounon P, Chiche J, Roux D, et al (2009) Hypoxia-induced autophagy is mediated through hypoxia-inducible factor induction of BNIP3 and BNIP3L via their BH3 domains. Mol Cell Biol 29: 2570–2581. 19273585 10.1128/MCB.00166-09 PMC2682037

[44] Lin MT, Kuo IH, Chang CC, Chu CY, Chen HY, et al (2008) Involvement of hypoxia-inducing factor-1alpha-dependent plasminogen activator inhibitor-1 up-regulation in Cyr61/CCN1-induced gastric cancer cell invasion. J Biol Chem 283: 15807–15815. 18381294 10.1074/jbc.M708933200 PMC3259638

[45] Kurokawa T, Miyamoto M, Kato K, Cho Y, Kawarada Y, et al (2003) Overexpression of hypoxia-inducible-factor 1 alpha(HIF-1 alpha) in oesophageal squamous cell carcinoma correlates with lymph node metastasis and pathologic stage. British Journal of Cancer 89: 1042–1047. 12966423 10.1038/sj.bjc.6601186 PMC2376949

[46] Whitney LK, Williams KJ, Stratford IJ, Lunt SJ, Brown L (2004) The role of hypoxia and the transcription factor HIF-1 in the development of metastasis. British Journal of Cancer 91: S50–S50.

[47] Bernhardt WM, Campean V, Kany S, Jurgensen JS, Weidemann A, et al (2006) Preconditional activation of hypoxia-inducible factors ameliorates ischemic acute renal failure. Journal of the American Society of Nephrology 17: 1970–1978. 16762988 10.1681/ASN.2005121302

[48] Philipp S, Jurgensen JS, Fielitz J, Bernhardt WM, Weidemann A, et al (2006) Stabilization of hypoxia inducible factor rather than modulation of collagen metabolism improves cardiac function after acute myocardial infarction in rats. European Journal of Heart Failure 8: 347–354. 16513418 10.1016/j.ejheart.2005.10.009

[49] Sen Banerjee S, Thirunavukkarasu M, Rishi MT, Sanchez JA, Maulik N, et al (2012) HIF-prolyl hydroxylases and cardiovascular diseases. Toxicol Mech Methods 22: 347–358. 22424133 10.3109/15376516.2012.673088

[50] Zhou YX, Huang YL (2009) Antiangiogenic effect of celastrol on the growth of human glioma: an in vitro and in vivo study. Chin Med J (Engl) 122: 1666–1673. 19719969

[51] Zhu H, Liu XW, Cai TY, Cao J, Tu CX, et al (2010) Celastrol acts as a potent antimetastatic agent targeting beta1 integrin and inhibiting cell-extracellular matrix adhesion, in part via the p38 mitogen-activated protein kinase pathway. J Pharmacol Exp Ther 334: 489–499. 20472666 10.1124/jpet.110.165654

[52] Kim Y, Kang H, Jang SW, Ko J (2011) Celastrol inhibits breast cancer cell invasion via suppression of NF-kB-mediated matrix metalloproteinase-9 expression. Cell Physiol Biochem 28: 175–184. 21865725 10.1159/000331729

[53] Lerman OZ, Greives MR, Singh SP, Thanik VD, Chang CC, et al (2010) Low-dose radiation augments vasculogenesis signaling through HIF-1-dependent and -independent SDF-1 induction. Blood 116: 3669–3676. 20631377 10.1182/blood-2009-03-213629

[54] Harada H, Kizaka-Kondoh S, Li G, Itasaka S, Shibuya K, et al (2007) Significance of HIF-1-active cells in angiogenesis and radioresistance. Oncogene 26: 7508–7516. 17563752 10.1038/sj.onc.1210556

[55] Hamacher-Brady A, Brady NR, Logue SE, Sayen MR, Jinno M, et al (2007) Response to myocardial ischemia/reperfusion injury involves Bnip3 and autophagy. Cell Death Differ 14: 146–157. 16645637 10.1038/sj.cdd.4401936

[56] Deng YN, Shi J, Liu J, Qu QM (2013) Celastrol protects human neuroblastoma SH-SY5Y cells from rotenone-induced injury through induction of autophagy. Neurochem Int 63: 1–9. 23619395 10.1016/j.neuint.2013.04.005

[57] Lee HW, Jang KS, Chun KH (2014) Celastrol inhibits gastric cancer growth by induction of apoptosis and autophagy. BMB Rep. 10.5483/BMBRep.2014.47.12.069 PMC4345515 24667175

[58] Kannaiyan R, Manu KA, Chen L, Li F, Rajendran P, et al (2011) Celastrol inhibits tumor cell proliferation and promotes apoptosis through the activation of c-Jun N-terminal kinase and suppression of PI3 K/Akt signaling pathways. Apoptosis 16: 1028–1041. 21786165 10.1007/s10495-011-0629-6

